# Supplementary figures and images for: An in vitro model for vitamin A transport across the human blood–brain barrier
Source: eLife. 2023 Nov 7;12:RP87863. doi: 10.7554/eLife.87863 (PMC10629827; doi:10.7554/eLife.87863)

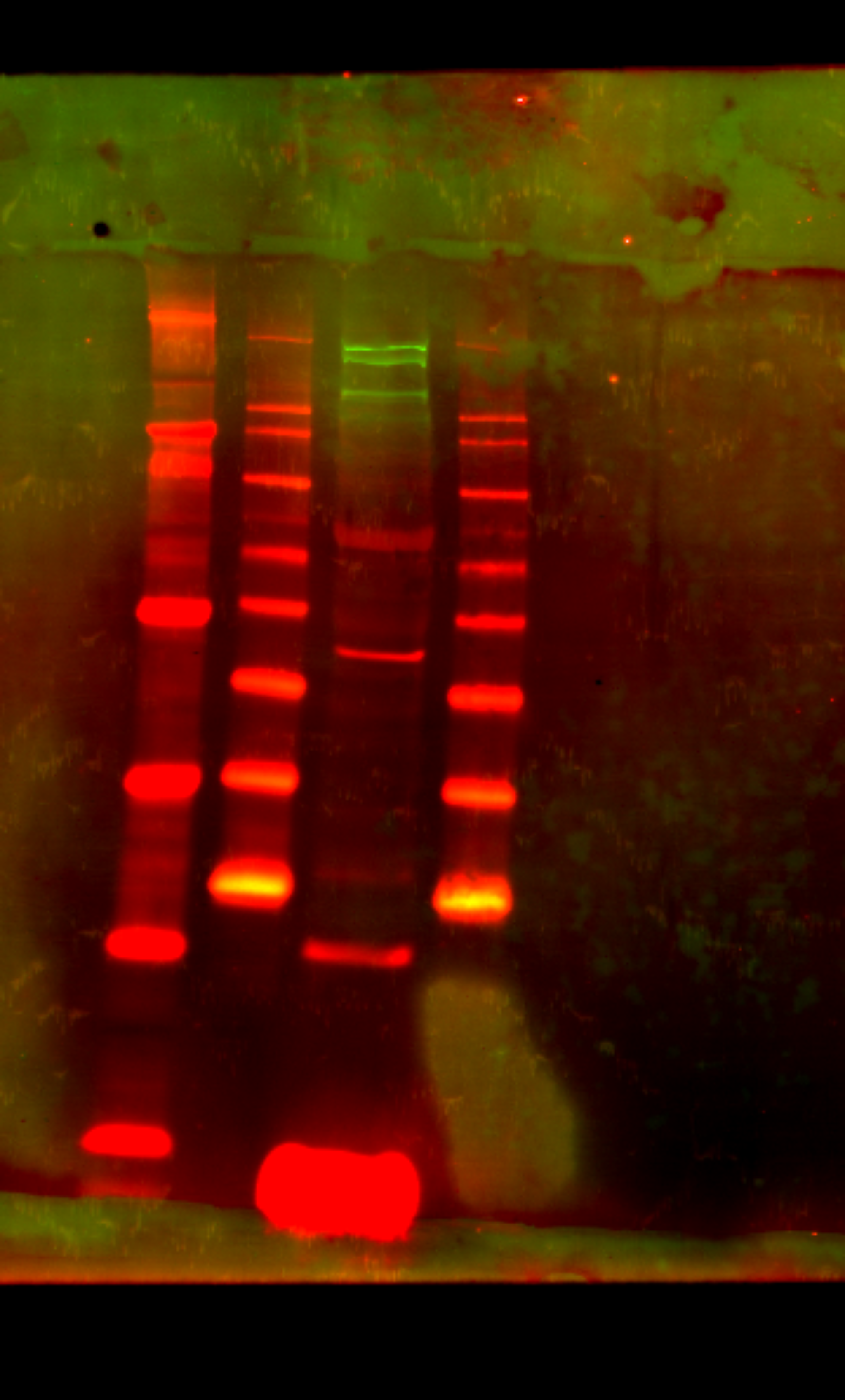

Supplement: Figure 2—source data 1. [file elife-87863-fig2-data1.tif]

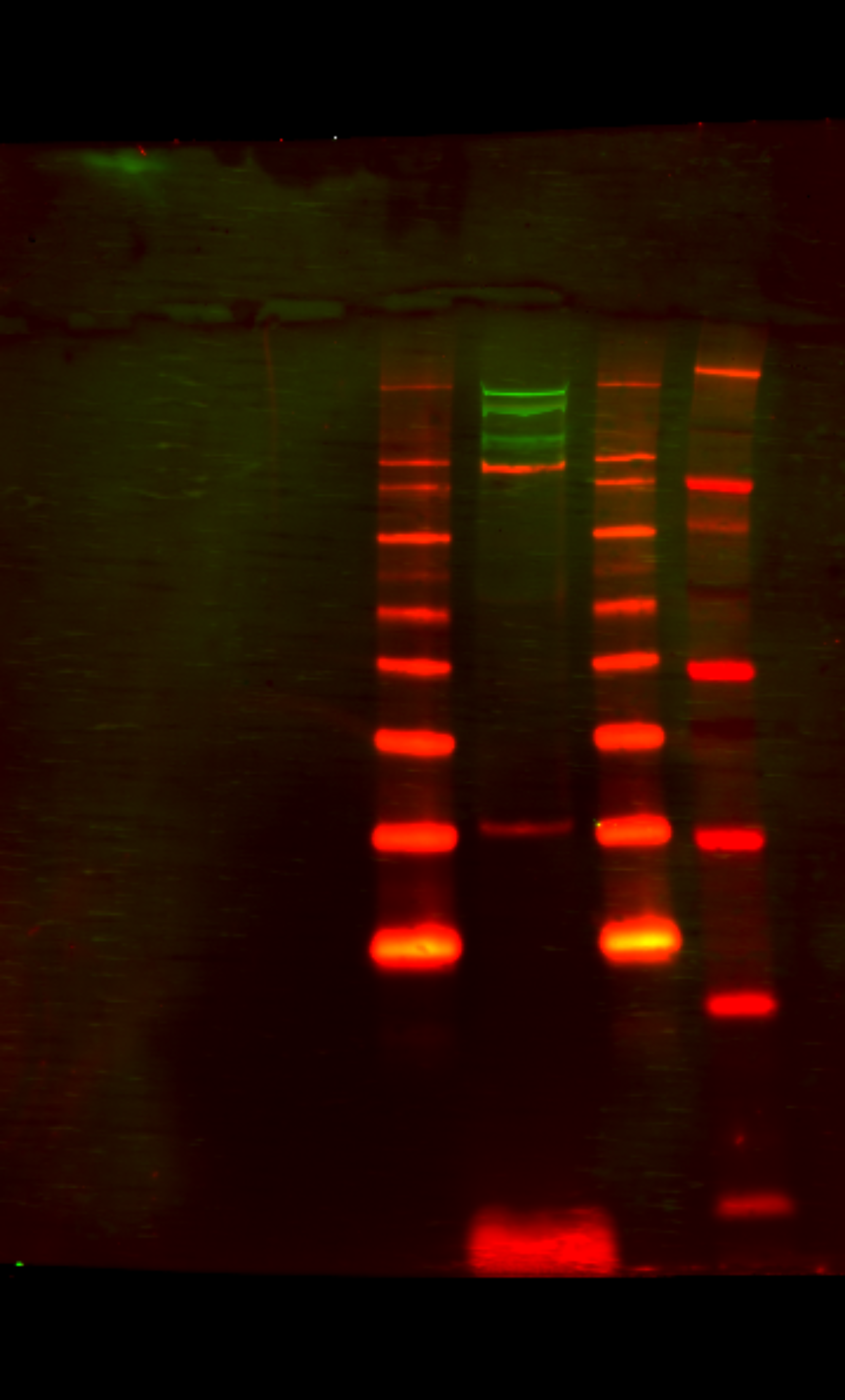

Supplement: Figure 2—source data 2. [file elife-87863-fig2-data2.tif]

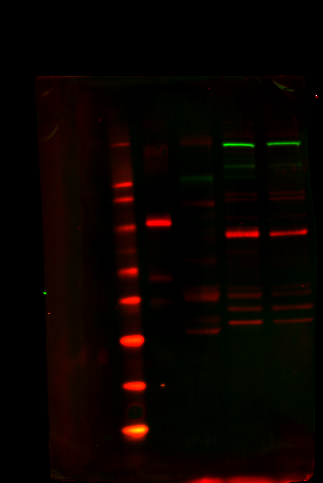

Supplement: Figure 2—source data 3. [file elife-87863-fig2-data3.tif]
