# Supplementary material for: An in vitro model for vitamin A transport across the human blood–brain barrier
Source: eLife. 2023 Nov 7;12:RP87863. doi: 10.7554/eLife.87863 (PMC10629827; doi:10.7554/eLife.87863)
Supplement: Figure 2—source data 4. [file elife-87863-fig2-data4.pdf]

CRBP1

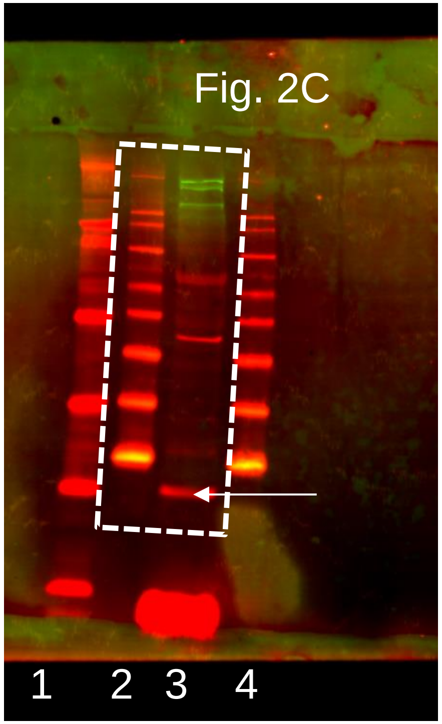

LRAT

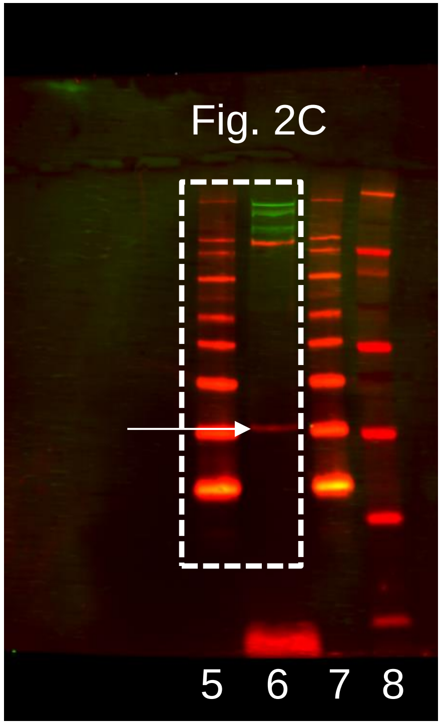

STRA6

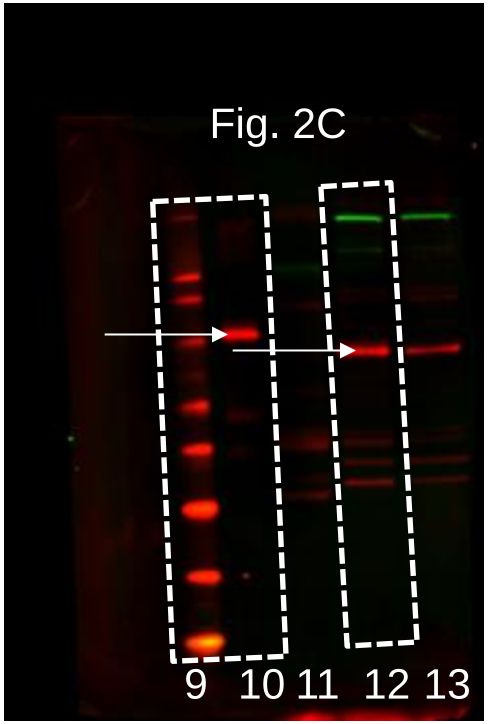

Lane	Sample
1	Ladder
2	Ladder
3	BMEC Lysate
4	Ladder
5	Ladder
6	BMEC Lysate
7	Ladder
8	Ladder
9	Ladder
10	Recombinant GST-STRA6
11	Schwann Cell Lysate (should not express STRA6)
12	BMEC Lysate
13	BMEC Lysate
