# Supplementary material for: An in vitro model for vitamin A transport across the human blood–brain barrier
Source: eLife. 2023 Nov 7;12:RP87863. doi: 10.7554/eLife.87863 (PMC10629827; doi:10.7554/eLife.87863)
Supplement: Supplementary file 1. — (a) Mass balances for 3H-ROH. (b) Mass balances for 14C-sucrose. (c) Mean apparent permeability of 3H-ROH and 14C-sucrose. (d) Maximum percentage of fluid phase ROH accumulated by cells. (e) Partitioning model fits for BMEC free ROH accumulation. [file elife-87863-supp1.docx]

**Supplementary File 1**

An in vitro model for vitamin A transport across the human blood-brain barrier

*Chandler B. Est^2^ and Regina M. Murphy^1^*

Department of Chemical and Biological Engineering, University of Wisconsin – Madison

1415 Engineering Dr., Madison, WI 53706

^1^ Corresponding author. *E-mail address:* [regina.murphy@wisc.edu](mailto:regina.murphy@wisc.edu)

^2^ Current address: Division of Endocrinology, Metabolism and Lipid Research, Washington University School of Medicine, 660 South Euclid Avenue, St. Louis, MO, 63110

**Supplementary file 1a: Mass balances for ^3^H-ROH**

| Sample | Replicate | Apical Counts  x 10^-3^ (t0) | Apical Counts  x 10^-3^ (t60) | Basolateral Counts  x 10^-3^  (t60) | Lysate Counts x 10^-3^ (t60) | Missing Counts x 10^-3^ (t60) | % Missing |
| --- | --- | --- | --- | --- | --- | --- | --- |
| 0.1 µM Free ROH | 1 | 227.22 | 218.21 | 4.05 | 7.33 | (2.38) | -1.0% |
|  | 2 | 235.00 | 218.08 | 4.77 | 7.26 | 4.89 | 2.1% |
|  | 3 | 238.90 | 218.61 | 4.68 | 8.23 | 7.37 | 3.1% |
|  | 4 | 233.22 | 219.94 | 4.98 | 8.83 | (0.52) | -0.2% |
| 0.4 µM Free ROH | 1 | 326.57 | 295.66 | 7.81 | 13.70 | 9.40 | 2.9% |
|  | 2 | 326.87 | 291.44 | 8.98 | 13.43 | 13.02 | 4.0% |
|  | 3 | 327.87 | 292.72 | 9.28 | 13.44 | 12.43 | 3.8% |
|  | 4 | 333.21 | 300.92 | 10.26 | 15.28 | 6.75 | 2.0% |
| 2 µM Free ROH | 1 | 1,552.98 | 1,171.46 | 58.02 | 164.43 | 159.07 | 10.2% |
|  | 2 | 1,537.16 | 1,161.60 | 57.20 | 171.93 | 146.43 | 9.5% |
|  | 3 | 1,545.32 | 1,206.34 | 63.14 | 169.63 | 106.21 | 6.9% |
|  | 4 | 1,547.17 | 1,173.19 | 65.65 | 184.56 | 123.77 | 8.0% |
| ROH-RBP | 1 | 1,799.84 | 1,675.54 | 60.68 | 49.51 | 14.11 | 0.8% |
|  | 2 | 1,840.65 | 1,688.63 | 62.44 | 53.72 | 35.85 | 1.9% |
|  | 3 | 1,848.14 | 1,681.03 | 62.71 | 54.53 | 49.87 | 2.7% |
|  | 4 | 1,819.80 | 1,680.83 | 66.05 | 63.90 | 9.01 | 0.5% |
| ROH-muRBP | 1 | 1,534.63 | 1,427.66 | 59.51 | 67.42 | (19.96) | -1.3% |
|  | 2 | 1,594.58 | 1,436.75 | 59.61 | 70.31 | 27.91 | 1.8% |
|  | 3 | 1,596.38 | 1,457.58 | 62.00 | 83.02 | (6.22) | -0.4% |
|  | 4 | 1,555.79 | 1,441.91 | 62.05 | 74.77 | (22.95) | -1.5% |
| ROH-RBP-TTR^[[1]](#footnote-1)^ | 1 | 843.25 | 755.65 | 38.36 | 23.94 | 25.29 | 3.0% |
|  | 2 | 842.47 | 798.55 | 36.95 | 27.37 | (20.40) | -2.4% |
|  | 3 | 826.47 | 778.79 | 36.47 | 23.65 | (12.45) | -1.5% |
|  | 4 | 842.34 | 795.80 | 36.42 | 23.15 | (13.03) | -1.5% |
| ROH-RBP-muTTR^1^ | 1 | 717.36 | 681.12 | 35.87 | 16.56 | (16.19) | -2.3% |
|  | 2 | 724.06 | 679.06 | 36.40 | 15.12 | (6.52) | -0.9% |
|  | 3 | 736.88 | 683.53 | 37.13 | 15.02 | 1.20 | 0.2% |
|  | 4 | 731.62 | 679.60 | 37.71 | 15.34 | (1.03) | -0.1% |

**Supplementary file 1b: Mass balances for ^14^C-sucrose**

| Sample | Replicate | Apical Counts x 10^-3^ (t0) | Apical Counts x 10^-3^ (t60) | Basolateral Counts  x 10^-3^  (t60) | Lysate Counts x 10^-3^ (t60) | Missing Counts x 10^-3^ (t60) | % Missing |
| --- | --- | --- | --- | --- | --- | --- | --- |
| 0.1 µM Free ROH | 1 | 590.78 | 603.22 | 2.48 | 6.70 | (21.62) | -3.7% |
|  | 2 | 608.26 | 602.21 | 2.28 | 5.86 | (2.09) | -0.3% |
|  | 3 | 612.02 | 600.11 | 2.75 | 8.73 | 0.44 | 0.1% |
|  | 4 | 604.47 | 599.49 | 2.75 | 11.88 | (9.65) | -1.6% |
| 0.4 µM Free ROH | 1 | 659.44 | 657.39 | 2.77 | 5.84 | (6.56) | -1.0% |
|  | 2 | 665.84 | 647.03 | 4.25 | 4.32 | 10.24 | 1.5% |
|  | 3 | 666.92 | 645.73 | 3.21 | 3.94 | 14.04 | 2.1% |
|  | 4 | 666.63 | 663.05 | 3.63 | 5.43 | (5.47) | -0.8% |
| 2 µM Free ROH | 1 | 649.77 | 631.68 | 3.35 | 4.39 | 10.35 | 1.6% |
|  | 2 | 645.77 | 633.23 | 3.33 | 5.15 | 4.05 | 0.6% |
|  | 3 | 646.95 | 634.52 | 3.15 | 4.55 | 4.73 | 0.7% |
|  | 4 | 648.13 | 628.08 | 3.23 | 5.64 | 11.19 | 1.7% |
| ROH-RBP | 1 | 663.97 | 678.58 | 4.36 | 5.13 | (24.11) | -3.6% |
|  | 2 | 663.64 | 668.09 | 5.16 | 6.18 | (15.79) | -2.4% |
|  | 3 | 670.71 | 667.15 | 4.55 | 5.38 | (6.37) | -0.9% |
|  | 4 | 662.58 | 680.76 | 4.97 | 9.01 | (32.17) | -4.9% |
| ROH-muRBP | 1 | 639.32 | 664.00 | 5.06 | 5.32 | (35.06) | -5.5% |
|  | 2 | 651.86 | 660.14 | 4.83 | 5.11 | (18.21) | -2.8% |
|  | 3 | 654.17 | 662.85 | 5.01 | 5.14 | (18.83) | -2.9% |
|  | 4 | 642.95 | 662.28 | 5.57 | 5.70 | (30.60) | -4.8% |
| ROH-RBP-TTR | 1 | 656.36 | 656.31 | 3.38 | 8.04 | (11.37) | -1.7% |
|  | 2 | 650.94 | 668.70 | 3.42 | 8.64 | (29.82) | -4.6% |
|  | 3 | 657.11 | 666.45 | 3.35 | 7.47 | (20.16) | -3.1% |
|  | 4 | 650.99 | 670.19 | 3.63 | 6.77 | (29.59) | -4.5% |
| ROH-RBP-muTTR | 1 | 602.46 | 619.32 | 2.77 | 5.59 | (25.21) | -4.2% |
|  | 2 | 598.84 | 620.44 | 3.22 | 4.95 | (29.77) | -5.0% |
|  | 3 | 611.35 | 616.41 | 3.03 | 5.52 | (13.61) | -2.2% |
|  | 4 | 606.02 | 614.02 | 2.68 | 5.15 | (15.83) | -2.6% |

**Supplementary file 1c: Mean apparent permeability of ^3^H-ROH and ^14^C-sucrose^[[2]](#footnote-2)^**

| Sample | ROH ${Pe}_{app}$  (10^-6^ cm/s) | Sucrose ${Pe}_{app}$  (10^-6^ cm/s) |
| --- | --- | --- |
| 0.1 µM Free ROH | 3.75 ± 0.39 | 0.43 ± 0.03 |
| 0.4 µM Free ROH | 4.65 ± 0.43 | 0.48 ± 0.04 |
| 2 µM Free ROH | 7.68 ± 0.53 | 0.58 ± 0.03 |
| ROH-RBP | 4.76 ± 0.19 | 0.69 ± 0.04 |
| ROH-muRBP | 5.44 ± 0.17 | 0.76 ± 0.06 |
| ROH-RBP-TTR | 6.56 ± 0.30 | 0.58 ± 0.03 |
| ROH-RBP-muTTR | 7.61 ± 0.13 | 0.55 ± 0.04 |

|  | $C_{V}=V_{D}\frac{S_{A,t}}{S_{D,t = 60 min}}$ | **Eq (S1)** |
| --- | --- | --- |
|  | ${Pe}_{app}=\frac{1}{A_{F}}m^{C_{V}}$ | **Eq (S2)** |

**Supplementary file 1d: Maximum percentage of fluid phase ROH accumulated by cells**

| Sample | Maximum percentage accumulated |
| --- | --- |
| 0.1 µM Free ROH | 16% |
| 0.4 µM Free ROH | 19% |
| 2 µM Free ROH | 28% |
| ROH-RBP | 8% |
| ROH-muRBP | 21% |
| ROH-RBP-TTR | 9% |
| ROH-RBP-muTTR | 8% |

**Supplementary file 1e: Partitioning model fits for BMEC free ROH accumulation**

| Parameter | Eq 1. Fit | Eq 2. Fit |
| --- | --- | --- |
| $K_{p}$ (µM cell/ µM fluid) | 131 | 99 |
| $k_{1}$ (min^-1^) | 0.021 | 0.027 |
| ${K_{p}}^{*}$ (µM cell/ µM fluid) | N/A | 90 |
| ${k_{1}}^{*}$ (min^-1^) | N/A | 0.014 |
| $t_{lag}$ (min) | N/A | 0.1 µM Free ROH: N/A  0.4 µM Free ROH: 101  2 µM Free ROH: 7.8 |
| ${c_{cell}}^{*}$ (µM) | N/A | 36 |
| $\chi^{2}$ | 17,930 | 2,470 |
| Residual Sum of Squares (RSS) | 41,000 | 3,700 |
| Aikake Information Criteria (AIC) | 17,934 | 2,480 |

$\frac{c_{cell}}{c_{f}}=K_{p}\left[ 1-exp\left( -k_{1}t \right) \right]$ **Eq. 1**

$\frac{c_{cell}}{c_{f}}=K_{p}\left[ 1-exp\left( -k_{1}t \right) \right]+{K_{p}}^{*}\left[ 1-exp\left( -{k_{1}}^{*}\left( t-t_{lag} \right) \right) \right]$ **Eq. 2**

where

$$t_{lag}=\frac{-ln\left[ 1-\frac{{c_{cell}}^{*}}{K_{p}c_{f}} \right]}{k_{1}}$$

1. TTR and muTTR samples were prepared with a target ^3^H-ROH : unlabeled-ROH ratio of 1:40 in order to reduce the ethanol concentration required for 2X ^3^H-ROH-RBP stocks. Precipitation of RBP was observed in 2X stocks prepared for use at 1:20 ratios. This problem only affected TTR and muTTR preparations because ROH-RBP and ROH-muRBP stocks could be prepared at 1X. [↑](#footnote-ref-1)
2. ${Pe}_{app}$ is the mean apparent permeability (*N = 4 replicates*) of the BMEC monolayer and Transwell filter combined calculated by **Eq (S2)**, where $A_{F}$ is the Transwell filter area and $m^{C_{V}}$is the fitted linear slope of the clearance volume ($C_{V}$) as a function of time. For samples displaying a lag-phase, slope was calculated only from the linear segment. $C_{V}$ values were calculated by **Eq (S1)**, where $V_{D}$ is the volume of the donor chamber and $S_{A,t}$ and $S_{D,t = 60 min}$ are the CPM signal in the acceptor chamber at time $t$ and the CPM signal in the donor chamber at time $t=60 min$, respectively. [↑](#footnote-ref-2)
